# Obituary

**Published:** 1873-04-01

**Authors:** 


### Obituary.

Death.—At a meeting of the Senior Class of the Chicago Medical College, the following resolutions were adopted:

*Whereas*, A mysterious Providence has removed from our midst our beloved classmate and brother, Mr. B. G. Tweed, who so soon would have finished his long and arduous pupilage and entered the active ranks of our profession,

*Resolved*, That in his death the Senior Class has sustained the loss of one of its most active and brilliant members, and the medical profession an able and enthusiastic supporter. He was a student of rare talent and promise. He was a Christian in principle and practice, zealous, gentle, and manly.

*Resolved*, That we tender to his bereaved mother and family our warmest sympathy in their great affliction.

*Resolved*, That a copy of these resolutions be sent to the bereaved family, and also to the medical journals.

J. S. McCord,
D. A. K. Steele,
Dan'l Lord,
*Committee.*

